# Supplementary material for: Preparation of a PVA/Chitosan/Glass Fiber Composite Membrane and the Performance in CO2 Separation
Source: Membranes (Basel). 2022 Dec 28;13(1):36. doi: 10.3390/membranes13010036 (PMC9863650; doi:10.3390/membranes13010036)
Supplement: Supplementary file 1 [file membranes-13-00036-s001.zip › membranes-2118324-supplementary.pdf]

Preparation of a PVA/Chitosan/Glass Fiber Composite Membrane and the Performance in CO_2 Separation

Yunwu Yu [1,*], Chunyang Xie [1], Yan Wu [1], Peng Liu [1], Ye Wan [1], Xiaowei Sun [1], Lihua Wang [1] and Yinan Zhang [2]

[1] School of Materials Science and Engineering, Shenyang Jianzhu University, Shenyang 110168, China
[2] Research Center for Nanotechnology, Changchun University of Science and Technology, Changchun 130022, China
* Correspondence: yuyunwu@sjzu.edu.cn

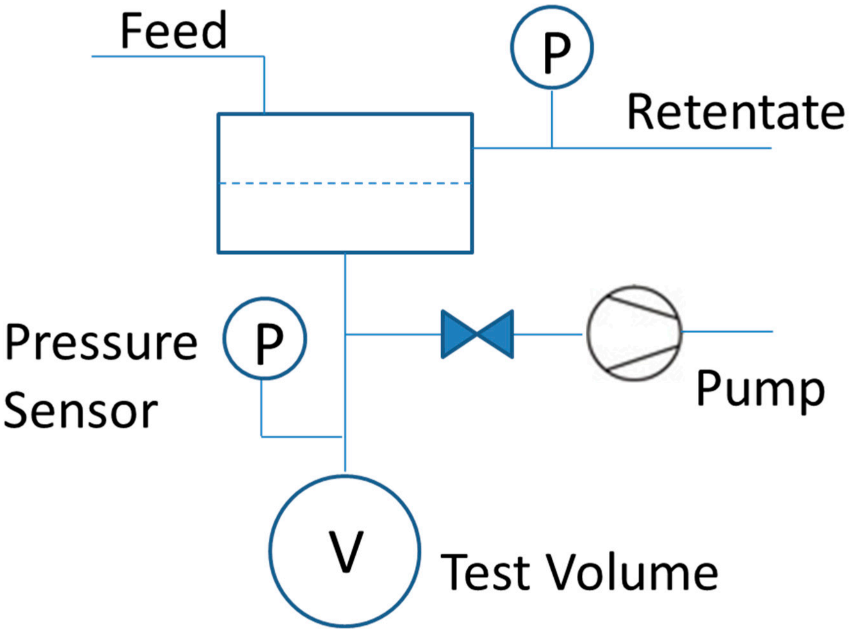

Figure S1. General device setup of a constant volume/variable pressure method test system.

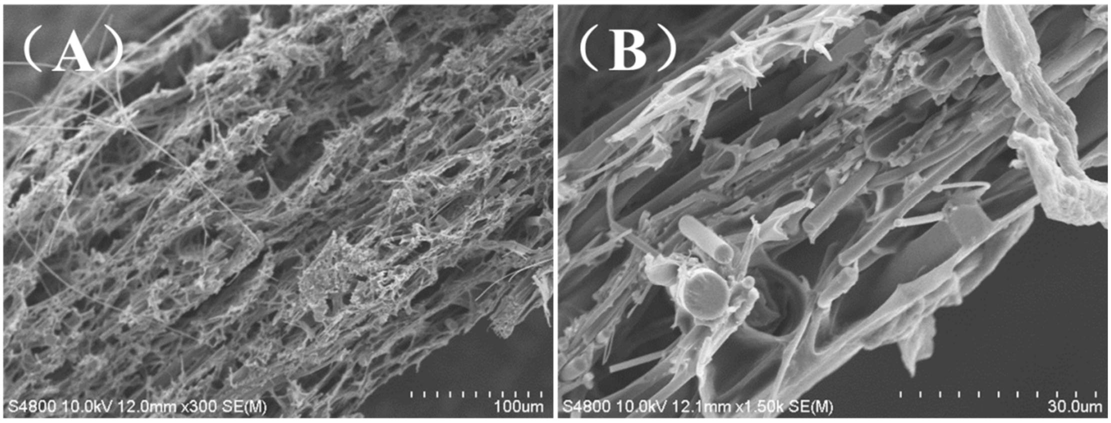

Figure S2. The morphology of of the membrane cross section. (A): CS4, (B): CS6.
